# The association between physical activity and lung function in adolescents: a cross-sectional NHANES study

**DOI:** 10.3389/fmed.2025.1538221

**Published:** 2025-06-03

**Authors:** Xiaoyuan Chen, Jinhai Ma, Linghua Dong, Yan Chen, Keke Chen

**Affiliations:** ^1^Department of Pediatrics, General Hospital of Ningxia Medical University, Yinchuan, China; ^2^Department of Respiratory and Critical Care Medicine, General Hospital of Northern Theater Command, Shenyang, China; ^3^Department of Clinical Medicine, Jiaxing University, Jiaxing, China

**Keywords:** physical activity, lung function, NHANES, adolescents, high-intensity exercise

## Abstract

**Background:**

This study investigates the relationship between physical activity (PA) and lung function in adolescents using data from the National Health and Nutrition Examination Survey (NHANES) from 2007 to 2012. While physical activity is known to improve respiratory health, its impact on specific lung function parameters in adolescents, particularly across different activity intensities, remains underexplored.

**Methods:**

We analyzed the effects of varying intensities of PA on lung function parameters—specifically Forced Vital Capacity (FVC), Forced Expiratory Volume in 1 s (FEV₁), and Forced Expiratory Flow (FEF)—in a sample of 896 adolescents aged 12–19 years. Linear regression analyses were employed to examine the relationship between PA duration and lung function, adjusting for potential confounders. The sample was stratified by intensity of PA (low, moderate, and high) to assess differential impacts on lung function.

**Results:**

The analysis revealed significant improvements in lung function with increased PA duration, with high-intensity PA showing the most pronounced effects. Specifically, high-intensity PA was associated with a 2.0 (95% CI: 0.43, 3.5) increase in FVC and a 2.0 (95% CI: 0.74, 3.4) increase in FEV₁ per unit increase in activity. Moderate-intensity PA also demonstrated significant improvements in lung function, though to a lesser extent. Stratified analyses identified significant associations within certain racial subgroups, with Mexican American and Other Hispanic adolescents more likely to maintain normal lung function.

**Conclusion:**

These findings highlight the importance of regular physical activity, particularly high-intensity exercise, in enhancing lung function during adolescence. The results emphasize the need for targeted public health interventions to encourage PA, especially in racially diverse populations. Further longitudinal studies are needed to evaluate the long-term effects of PA on lung health and to establish causal relationships.

## 1 Introduction

Lung function is a vital indicator of respiratory health, especially during childhood, when the respiratory system undergoes critical developmental stages (1). Optimal lung function during childhood not only supports immediate health but also contributes to long-term well-being, influencing susceptibility to chronic respiratory diseases later in life (2). Regular physical activity (PA) has been shown to play a crucial role in enhancing lung function by improving lung capacity and respiratory muscle strength (3, 4). Despite the established health benefits of PA, its direct relationship with lung function in children remains underexplored, with limited large-scale evidence addressing this connection (5).

Global health guidelines emphasize the importance of PA for children, recommending at least 60 min of moderate-to-vigorous physical activity (MVPA) daily (6, 7). Such activity has been linked to improvements in spirometry outcomes, including forced vital capacity (FVC) and forced expiratory volume in 1 s (FEV_1_), which are widely recognized measures of lung function (8, 9). Although the findings suggest that physical activity (PA) may have potential benefits for respiratory health, the effects of various types, intensities, and durations of PA on lung function in children remain insufficiently clarified (10). Furthermore, children’s participation in PA is frequently influenced by demographic factors, environmental conditions, and seasonal variations, which further complicate the relationship between PA and lung function (11).

Existing studies examining the relationship between physical activity and lung function in children often focus on small, localized populations or specific contexts, which limits the generalizability of their findings (1, 12, 13). Moreover, while previous research has provided valuable insights into the benefits of PA, it has not systematically explored the variations in these benefits across different subgroups or settings (14–16). To address these gaps, it is crucial to obtain robust data from large, nationally representative cohorts to gain a more comprehensive understanding of the complex relationship between PA and lung function (8, 17).

This study aims to examine the association between physical activity and lung function in children using data from the National Health and Nutrition Examination Survey (NHANES) for the years 2007–2012. Specifically, it investigates how varying levels and types of PA influence key lung function parameters, such as FVC and FEV_1_. Additionally, this study seeks to explore potential variations in these associations based on demographic factors, such as age and sex. We hypothesize that regular PA has a positive effect on lung function, with variations influenced by the type and intensity of the activity. By utilizing a large, nationally representative dataset, this research offers a comprehensive assessment of the relationship between PA and lung function in children. The findings are expected to provide valuable evidence for public health initiatives aimed at enhancing respiratory health through physical activity and for developing targeted interventions to optimize children’s lung function.

## 2 Materials and methods

### 2.1 Data source

The U.S. National Health and Nutrition Examination Study is a series of nationally representative cross-sectional studies sponsored and approved by the National Center for Health Statistics (NCHS). The study, conducted every 2 years by the Centers for Disease Control and Prevention (CDC), collects health and nutritional data from the U.S. population using a complex, stratified sampling design to ensure representative samples of non-institutionalized civilians. Participants undergo detailed in-home interviews, physical examinations, and blood specimen collections at specially equipped mobile examination centers. All participants provided written informed consent, and the study was approved by the Institutional Ethics Review Board of NCHS. The data are publicly available and anonymized, and the Ethical Committee and Institutional Review Board Committee of Xinhua Hospital exempted the study from ethics review (18). The investigation adheres to the principles outlined in the Declaration of Helsinki. Detailed descriptions and protocols of the NHANES study can be found online.^1^

### 2.2 Study population

**Figure 1:**
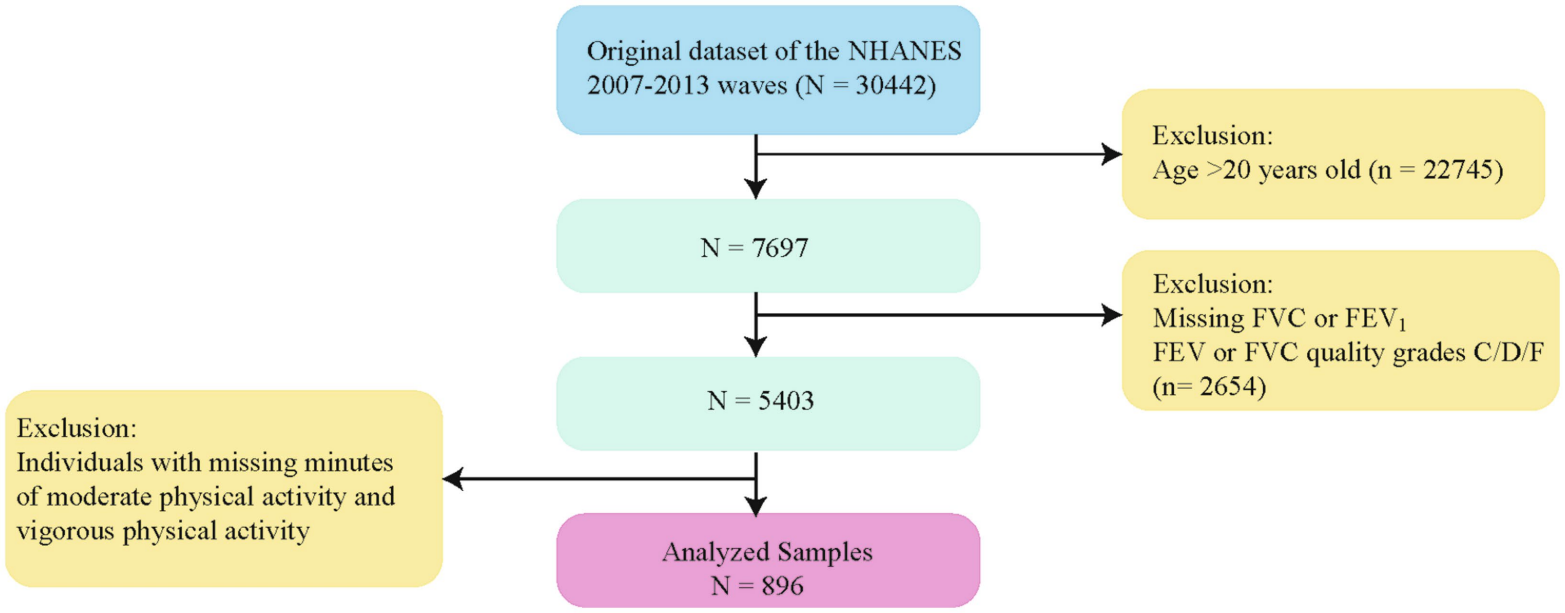
Flowchart for selecting analyzed participants. FEV₁, forced expiratory volume in one second; FVC, forced vital capacity; NHANES, National Health and Nutrition Examination Survey.

This study utilized data from three two-year cycles of the NHANES conducted between 2007 and 2012. The dataset included comprehensive information on demographics, physical examinations, medical history, dietary patterns, comorbidities, and laboratory measurements. Data from this period were selected because lung function test results were only comprehensively available for the 2007–2012 cycles. An initial total of 30,442 participants was identified, and the following exclusion criteria were applied:

(1) aged>20 years old (*n* = 22,745); (2) Individuals with conditions potentially interfering with lung function testing, including current chest pain, restricted forced expiration, dependence on supplemental oxygen, recent surgery, myocardial infarction, stroke, tuberculosis exposure, or a history of hemoptysis; (3) Participants missing key lung function test results, such as FEV₁ or FVC, or those with low-quality lung function test data (quality grades of C, D, or F) (*n* = 2,654);(4) Participants without recorded data on time spent in moderate or vigorous physical activity during recreational activities. After applying these criteria, the final study population consisted of adolescents aged 12–19 years (19) (*n* = 896), providing a nationally representative sample for analysis (Figure 1).

### 2.3 Assessment of lung function parameters

Lung function tests were performed using the Ohio 822/827 dry rolling seal spirometer, following the standards established by the American Thoracic Society (ATS) and the European Respiratory Society (ERS) (20). Key variables analyzed in this study included FEV₁, FVC, and the FEV₁/FVC ratio. The quality of the tests was graded from A to F according to ATS/ERS criteria: Grades A and B indicate tests that meet or exceed the standards, Grade C indicates data that may be acceptable, while Grades D to F are considered potentially invalid. To ensure data reliability, this study included only test results with FEV₁ and FVC graded as A or B, excluding data with Grades C, D, or F (21).

### 2.4 Assessment of physical activity data

Physical activity data were collected using the WHO Global Physical Activity Questionnaire as part of the NHANES surveys conducted between 2007 and 2016. The primary measure was the total minutes of moderate or vigorous recreational activities performed per week (22, 23). Moderate-intensity activities included exercises that slightly increased breathing or heart rate, such as brisk walking, cycling, swimming, or golfing. Vigorous-intensity activities referred to exercises that significantly elevated breathing or heart rate, such as running or playing basketball. For sensitivity analyses, additional evaluations incorporated total PA time, including occupational and transportation activities, to provide a comprehensive assessment of PA across all domains (24).

### 2.5 Statistical analysis

Lung function parameters and other continuous variables were expressed as mean ± standard deviation (SD), while categorical variables were summarized as frequencies or percentages. All analyses incorporated survey weights to account for the complex sampling design. The primary objective of this study was to investigate the relationship between physical activity levels and lung function indices. Linear regression analyses within the framework of GEE were employed to evaluate the associations of moderate- and vigorous-intensity recreational activities with lung function outcomes. Stratified analyses were performed by age, sex, and race to explore potential effect modifications. Additionally, restricted cubic spline models were used to assess nonlinear associations between moderate-intensity activity and lung function indices. Statistical analyses were conducted using R (25) software (version 4.3.3, http://www.R-project.org) and DecisionLinnc 1.0 software.^2^ A two-sided *p* < 0.05 was deemed statistically significant.

## 3 Results

### 3.1 Characteristics of the participants

The weighted distribution results (Table 1) summarize the demographic characteristics, physical examination findings, personal lifestyle history, and health-related data of the participants selected from the NHANES 2007–2012 survey. A total of 896 adolescents aged 12–19 years were included in the final analysis, representing a nationally representative sample.

**Table 1:** Baseline characteristics of participants (NHANES 2007–2012).

Variable	Level	0–30 min	30–60 min	> 60 min	*p*
*n*		92	104	700	
Age [mean (SD)]		13.31 (1.37)	14.53 (2.19)	15.31 (2.19)	<0.001
Sex *n* (%)	Female	58 (63.04)	56 (53.84)	273 (39.0)	<0.001
	Male	34 (36.96)	48 (46.16)	427 (61.0)	
Race *n* (%)	Mexican American	9(9.8)	14 (13.5)	70 (10.0)	<0.001
	Non-Hispanic Black	26(28.3)	12 (11.5)	69 (9.9)	
	Non-Hispanic White	47(51.1)	61 (58.7)	466 (66.6)	
	Other Hispanic	8(8.6)	6 (5.8)	40 (5.7)	
	Multi-Racial	2(2.2)	11 (10.5)	55 (7.8)	
BMI Kg/m^2^[mean (SD)]		20.47 (3.37)	23.79 (5.97)	23.36 (5.35)	0.047
MVPA. Time [mean (SD)]		23.85 (2.92)	45.09 (6.85)	164.26 (88.99)	<0.001
Asthma *n* (%)	Yes	8 (8.7)	19 (18.3)	133 (19.0)	0.052
	No	84 (91.3)	85 (81.7)	567 (81.0)	
FVC %pred [mean (SD)]		98.5 (12.3)	101.2 (13.5)	103.8 (14.1)	<0.001
FEV_1_%pred [mean (SD)]		97.8 (11.9)	100.5 (12.7)	102.9 (13.3)	<0.001
FEF %pred [mean (SD)]		96.2 (14.5)	99.1 (15.2)	101.5 (15.8)	<0.001

Comparisons across different physical activity durations revealed significant differences in various parameters. The mean age increased with longer activity durations (0–30 min: 13.31 ± 1.37 years; 30–60 min: 14.53 ± 2.19 years; >60 min: 15.31 ± 2.19 years; *p* < 0.001). Sex distribution showed an increasing proportion of males and a decreasing proportion of females with longer activity durations, with statistically significant differences (0–30 min: 36.96% male, 63.04% female; 30–60 min: 46.16% male, 53.84% female; >60 min: 61.0% male, 39.0% female; *p* < 0.001). Among racial groups, non-Hispanic White participants were the majority, with their proportion reaching 66.6% in the >60 min group, and significant differences were observed in racial distribution across groups (*p* < 0.001).

BMI was highest in the 30–60 min group (23.79 ± 5.97 kg/m^2^) compared to the other groups (0–30 min: 20.47 ± 3.37 kg/m^2^; >60 min: 23.36 ± 5.35 kg/m^2^), with statistically significant differences across groups (*p* = 0.047). Time spent in moderate-to-vigorous physical activity (MVPA) increased significantly with longer durations (0–30 min: 23.85 ± 2.92 min; 30–60 min: 45.09 ± 6.85 min; >60 min: 164.26 ± 88.99 min; p < 0.001). The prevalence of asthma showed a trend of increasing with longer activity durations (0–30 min: 8.7%; 30–60 min: 18.3%; >60 min: 19.0%), but the difference was not statistically significant (*p* = 0.052).

The %pred values for lung function parameters showed notable improvements with increased physical activity. FVC %pred increased from 98.5% in the 0–30 min group to 103.8% in the >60 min group, indicating that higher levels of physical activity are associated with lung volumes that are closer to or even exceed predicted values. Similarly, FEV₁ %pred increased from 97.8 to 102.9%, demonstrating that increased physical activity is linked to improvements in expiratory flow. FEF %pred also improved, rising from 96.2 to 101.5%, reflecting enhancements in mid-expiratory flow.

### 3.2 Association between physical activity and lung function

Table 2 illustrates that lung function indices, including FVC, FEV₁ and FEF parameters, consistently improved with longer physical activity durations. Relative to the 0–30 min group (reference), the 30–60 min group exhibited significant enhancements in lung function (*p* < 0.001). Even greater improvements were observed in the >60 min group (p < 0.001). Comparable trends were noted for FEF_25_, FEF_50_, and FEF_75_, with both the 30–60 min and >60 min groups demonstrating significant increases compared to the reference group (all *p* < 0.001).

**Table 2:** Association between physical activity duration and lung function.

Variable	FVC (mL) β (95%CI)^a^	FEV₁ (mL)	FEF (mL/s)	FEF_25_ (mL/s)	FEF_50_ (mL/s)	FEF_75_ (mL/s)
0-30 min	Reference	Reference	Reference	Reference	Reference	Reference
30-60 min	861.31 (654.47, 1068.15)*	791.17 (609.26, 973.08)*	867.02 (578.12, 1155.92)*	1517.28 (1011.70, 2022.86)*	953.72 (635.85, 1271.59)*	433.51 (289.04, 577.98)*
>60 min	1129.92 (992.23, 1267.61)*	1039.93 (912.10, 1167.76)*	1169.79 (949.76, 1389.82)*	2047.13 (1662.09, 2432.17)*	1286.77 (1044.65, 1528.89)*	584.89 (474.88, 694.90)*

FVC, forced vital capacity; FEV₁, forced expiratory volume in 1 s; FEF, forced expiratory flow (25–75%); FEF25, forced expiratory flow at 25% of vital capacity; FEF50, forced expiratory flow at 50% of vital capacity; FEF75, forced expiratory flow at 75% of vital capacity; CI, confidence interval. * Means *p* < 0.001.

a Adjusted for sex, age, BMI (body mass index), race and NHANES cycles.

### 3.3 Correlation between physical activity intensity and lung function

The regression analysis results (Table 3) demonstrate a positive correlation between both high- and moderate-intensity physical activities and lung function parameters. High-intensity activities showed particularly significant effects on FVC and FEV_1_, with *p*-values less than 0.01. The corresponding beta coefficients were 2.0 (95% CI: 0.43, 3.5) for FVC and 2.0 (95% CI: 0.74, 3.4) for FEV_1_. Similarly, moderate-intensity activities also exhibited positive associations, with beta coefficients of 2.4 (95% CI: 0.72, 4.0) for FVC and 1.5 (95% CI: 0.26, 2.8) for FEV_1_. Additionally, high-intensity activities were significantly associated with FEF (*β* = 3.1, 95% CI: 1.2, 5.0, *p* = 0.002), while moderate-intensity activities showed weaker and non-significant correlations (*p* = 0.4). For the FEV_1_/FEC ratio, no significant associations were observed in either intensity group. Overall, the tabulated results highlight that high-intensity physical activities exert a greater positive impact on lung function than moderate-intensity activities.

**Table 3:** Associations between physical activity intensity and lung function.

Activity intensity	FEV₁ (mL)		FVC (mL)		FEV_1_: FVC		FEF (mL/s)	
	*β* (95% CI)	*p*-value	*β* (95% CI)	*p*-value	*β* (95% CI)	*p*-value	*β* (95% CI)	*p*-value
Low intensity recreational activities	Reference		Reference		Reference		Reference	
High intensity recreational activities	2.0 (0.74, 3.4)	0.003	2.0 (0.43, 3.5)	0.013	0.000 (−0.011, 0.011)	0.2	3.1 (1.2, 5.0)	0.002
Moderate intensity recreational activities	1.5 (0.26, 2.8)	0.019	2.4 (0.72, 4.0)	0.006	0.000 (−0.007, 0.008)	0.06	0.76 (−1.1, 2.6)	0.4

**Figure 2:**
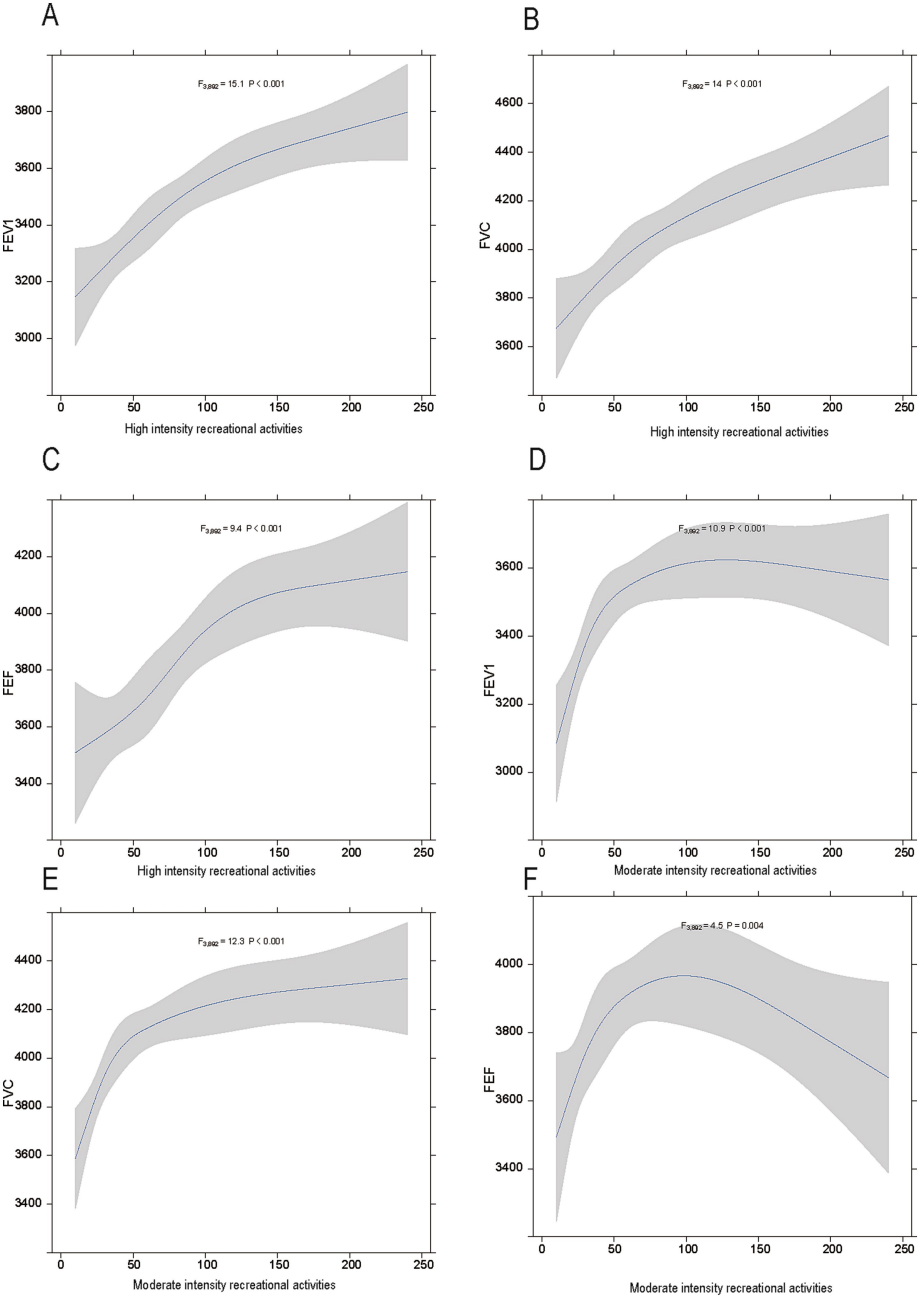
The relationship between physical activity intensity and lung function in adolescents. **(A–C)** show the associations between high-intensity physical activities and FEV_1_, FVC, and FEF, respectively. **(D–F)** depict the associations between moderate-intensity physical activities and FEV1, FVC, and FEF, respectively. Shaded areas represent 95% confidence intervals.

The visualized trends (Figure 2) further corroborate the influence of high- and moderate-intensity activities on lung function. In the high-intensity activity group, FEV_1_, FVC, and FEF demonstrated a significant upward trend with increasing activity duration (Figures 2A–C). Statistical analysis revealed that the changes in FEV_1_ were the most pronounced [*F* (3,892) = 15.1, *p* < 0.001], followed by significant changes in FVC [*F* (3,892) = 14, *p* < 0.001] and FEF [*F* (3,892) = 9.4, *p* < 0.001]. These findings suggest that high-intensity activities have a sustained and substantial impact on improving lung function over time.

For moderate-intensity activities (Figures 2D–F), FEV_1_, FVC, and FEF also showed proportional increases with activity duration, although the effects were less pronounced compared to high-intensity activities. Specifically, FEV_1_ [*F* (3,892) = 10.9, *p* < 0.001] and FVC [*F* (3,892) = 12.3, *p* < 0.001] demonstrated significant changes, while the improvement in FEF was relatively modest but still statistically significant [*F* (3,892) = 4.5, *p* = 0.004]. These visual results provide additional support for the regression analysis findings in the table and offer dynamic insights into the temporal effects of different physical activity intensities on the lung function.

**Figure 3:**
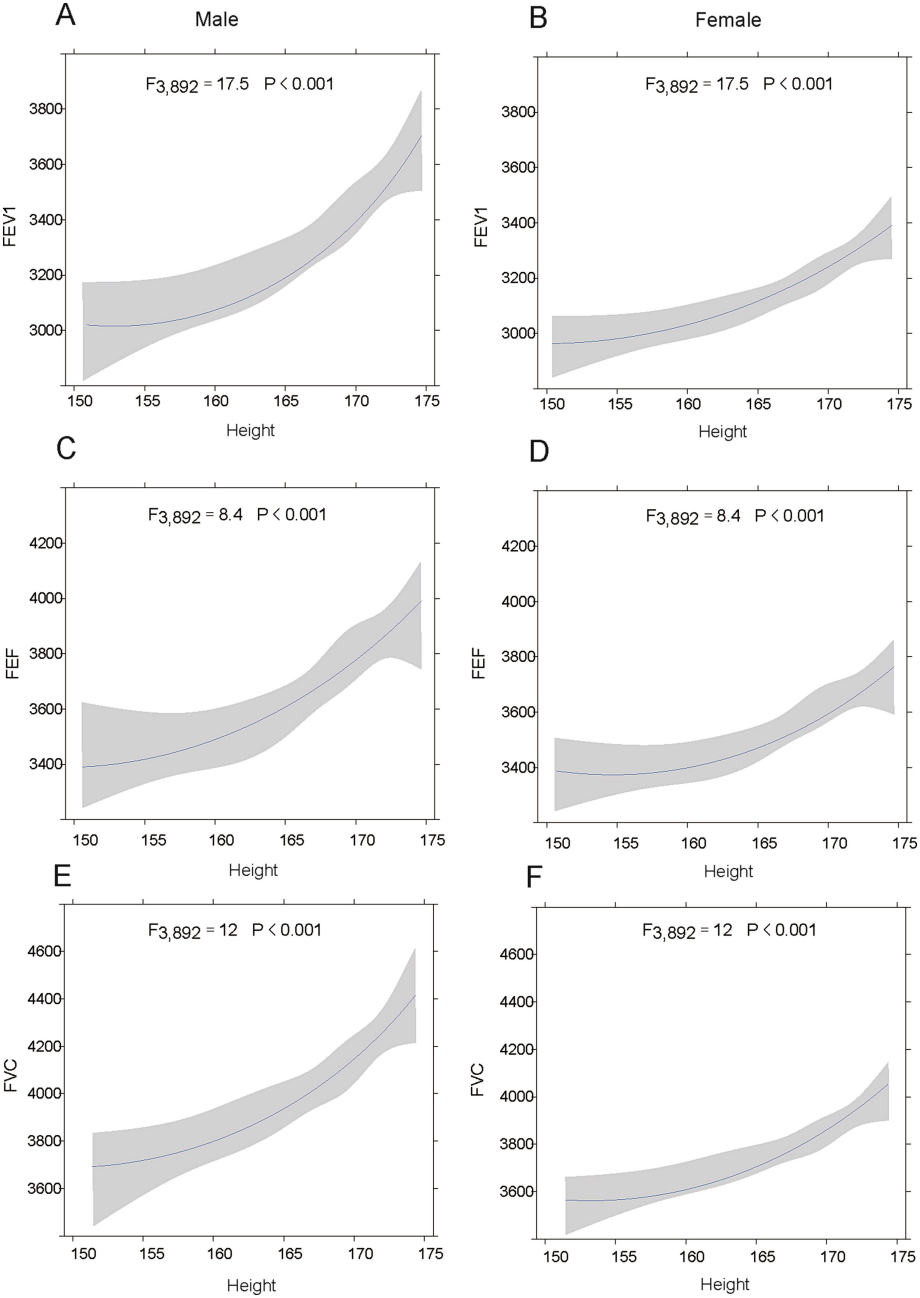
The relationship between height and lung function in adolescents. **(A-C)** show the associations between height of males and FEV_1_, FVC, and FEF, respectively. **(D–F)** depict the associations between height of females and FEV_1_, FVC, and FEF, respectively. Shaded areas represent 95% confidence intervals.

The Figure 3 describes the relationships between respiratory parameters (FEV_1_, FEF, and FVC) and height in males and females. Figures 3A–C show that taller males have higher FEV_1_, FEF and FVC values. Similarly, Figures 3D–F show that taller females have higher FEV_1_, FEF and FVC values. All models are statistically significant, indicating that height strongly affects these respiratory measures. While the correlations are similar, the absolute values and slopes differ slightly between sexes, reflecting anatomical and physiological differences. Thus, these results show that how crucial height is in interpreting respiratory function and considering sex differences for accurate assessment.

### 3.4 Stratified analysis of physical activity and lung function

**Figure 4:**
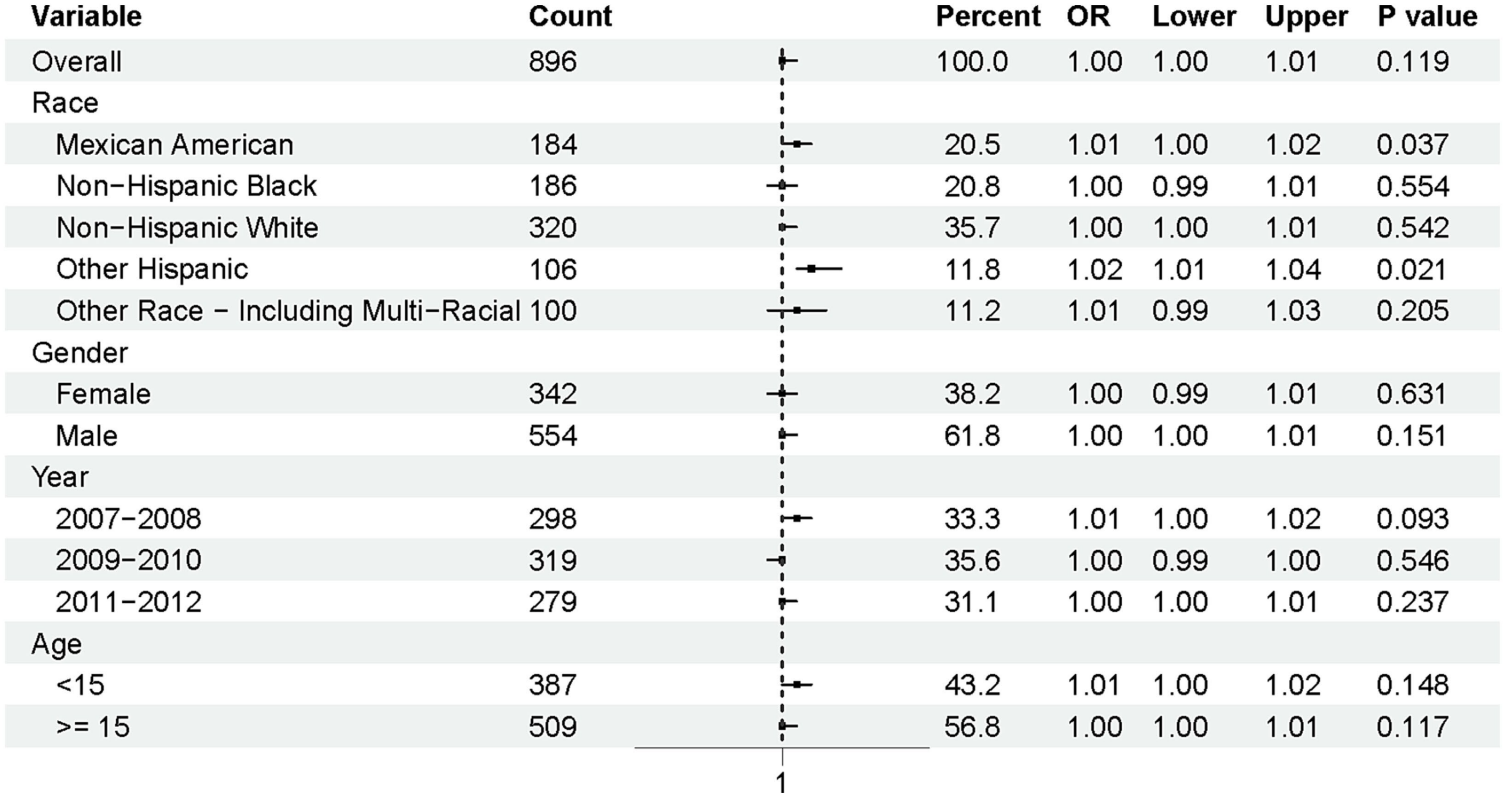
Stratified analysis of physical activity and lung function in adolescents.

To further explore the associations with normal lung function [defined as FEV_1_/FVC > 0.92 (26)], a subgroup analysis was conducted based on race, gender, year, and age (Figure 4). The overall odds ratio (OR) was 1.00 (95% CI: 1.00–1.01, *p* = 0.119), indicating no significant association at the population level. However, notable differences emerged across racial subgroups. Mexican Americans (OR = 1.01, 95% CI: 1.00–1.02, *p* = 0.037) and Other Hispanics (OR = 1.02, 95% CI: 1.01–1.04, *p* = 0.021) were significantly more likely to maintain normal lung function compared to other racial groups.

In contrast, no significant associations were observed for gender, year, or age groups. Specifically, the *p*-values for gender categories (female and male) were 0.631 and 0.151, respectively, suggesting minimal gender-based differences. Similarly, the time periods (2007–2008, 2009–2010, and 2011–2012) and age groups (<15 years and ≥15 years) did not show statistically significant trends, with *p*-values of 0.093 and 0.148 for the most relevant comparisons.

This figure illustrates the odds ratios (ORs) and 95% confidence intervals (CIs) for maintaining normal pulmonary function (FEV_1_/FVC > 0.92) among adolescents, stratified by race, gender, survey year, and age.

## 4 Discussion

In this study, we explored the relationship between physical activity (PA) and lung function in a nationally representative sample of 896 U.S. adolescents using data from the 2007-2012NHANES. Our findings indicate a statistically significant positive association between PA and key lung function parameters, including FVC, FEV₁ and FEF_25-75%_. Notably, high-intensity PA was associated with more pronounced benefits compared to moderate-intensity activities. Consistent with established physiological norms, height also emerged as a significant predictor, with taller adolescents exhibiting higher lung function values. Furthermore, subgroup analyses suggested potential ethnic disparities in the association between PA and the likelihood of maintaining normal lung function.

The observed positive association between PA and improved lung function in our adolescent cohort aligns with prior research. Longitudinal studies have linked higher childhood PA levels to better FVC and FEV₁ in later adolescence (27, 28). Regular PA has also been associated with a reduced incidence of restrictive spirometry patterns (8), corroborating our findings of enhanced lung capacity with increased activity. Furthermore, our results support evidence that high-intensity PA yields greater lung function improvements, consistent with the superior cardiopulmonary adaptations elicited by vigorous exercise (29). Indeed, our analysis of NHANES 2007–2012 data showed the most significant FEV₁ and FVC increases in the high-intensity PA group.

Contextualizing these findings within the U.S. adolescent population using NHANES data necessitates considering prevailing PA patterns. An analysis of NHANES 2007–2016 data identified significant sex and racial/ethnic disparities in PA levels among U.S. adolescents and young adults (30). This research highlighted lower PA engagement among females, as well as minority and low-income groups. While this study on PA patterns did not directly assess lung function, its findings from largely contemporaneous NHANES data offer a crucial context for the PA-related factors in our investigation. Building upon this, our study specifically links varying intensities of recreational PA to concrete lung function parameters (FVC, FEV₁), thereby complementing the existing NHANES-derived evidence.

Given the relative scarcity of NHANES-based studies quantifying PA’s precise effect sizes on adolescent FVC and FEV₁ improvements, our work provides such estimates. Specifically, high-intensity PA was associated with increases in FVC of 2.0 (95% CI: 0.43, 3.5) and in FEV₁ of 2.0 (95% CI: 0.74, 3.4) per unit increase in activity. PA has been linked to reduced restrictive ventilatory patterns (8). For further NHANES context, an analysis of 2007–2010 data on U.S. youths (aged 6–19 years) with asthma indicated that while their average lung function was lower, some maintained normal values (31). Although this NHANES study focused on asthmatics, it provides a useful, contemporaneous benchmark for adolescent lung function. Building on such NHANES-based insights, our study offers novel quantitative estimates of FVC and FEV₁ improvements related to specific PA durations and intensities in a broader adolescent population.

The mechanisms through which physical activity influences lung function is multifaceted. Physical activity enhances the strength of respiratory muscles and improves lung ventilation capacity (32–34). In children, improvements in lung function may be associated with increased lung compliance and elasticity, which directly influence airflow and the efficiency of alveolar ventilation (35–37). High-intensity exercise can stimulate adaptive responses in the respiratory system, increasing lung ventilation volume, which contributes to improvements in FEV_1_ and FVC (29, 38, 39). This aligns with the results of our study, where the most significant increases in FEV_1_ and FVC were observed in the high-intensity activity group. By enhancing the synergy between the respiratory system and muscles, physical activity improves the ability of the lungs to take in oxygen and increases overall lung capacity (40).

This study revealed that high-intensity PA confers superior improvements in lung function parameters compared to moderate-intensity PA. This finding is substantially supported by evidence linking high-intensity exercise to enhanced cardiorespiratory fitness (CRF) and favorable physiological adaptations. Improved CRF, particularly achieved through high-intensity training, profoundly modulates positive health outcomes and robustly supports the efficacy of such training in enhancing lung function (41). High-intensity training also effectively improves peak oxygen uptake (VO2 peak), a key indicator of aerobic fitness. Furthermore, even brief daily sessions (e.g., 5–10 min) of vigorous activities like running significantly reduce all-cause and cardiovascular mortality, suggesting broad physiological benefits from high-intensity PA that likely extend to respiratory health (42, 43).

Despite the more prominent lung function benefits of high-intensity PA, moderate-intensity PA also holds distinct value, primarily mediated through its anti-inflammatory effects, which can mitigate airway inflammation and improve lung compliance. Aerobic training in asthmatic patients, for instance, reduced airway inflammation markers, such as sputum eosinophils and fractional exhaled nitric oxide (FeNO) (44). Multiple mechanisms for exercise-induced anti-inflammatory actions, including favorable cytokine modulation (e.g., increased IL-10, inhibited TNF-*α*) and visceral fat reduction, have been detailed (45). Moreover, higher PA levels were associated with an attenuated decline in lung function and improved health status in COPD patients (46). Collectively, these anti-inflammatory and protective mechanisms offer a plausible explanation for the positive FEV1 and FVC trends observed with moderate-intensity PA in our study, likely by reducing underlying low-grade inflammation and enhancing airway reactivity.

Additionally, this study identified height as an important factor influencing lung function, especially among males. Taller adolescents demonstrated higher FEV₁, FVC, and FEF values, likely due to larger thoracic cavities and greater lung volumes, as noted by others (37). This anatomical advantage may amplify the benefits of PA, particularly in males, who generally possess greater muscle mass and respiratory efficiency. The observed differences in lung function by height highlight the need for tailored exercise programs that consider both anatomical factors and the intensity of physical activity.

Subgroup analysis revealed a higher likelihood of maintaining normal lung function among Mexican American and Other Hispanic adolescents. This observation is notable given established NHANES-derived literature on baseline lung function differences across U.S. racial and ethnic groups. For instance, foundational NHANES III (1988–1994) research established spirometric reference values for diverse populations aged 8–80, demonstrating inherent variations; notably, African Americans exhibited lower FVC and FEV₁ values than Caucasians and Mexican Americans, even after height adjustment (47). While our study focused on factors influencing the *maintenance* of normal lung function rather than baseline values themselves, these documented disparities provide crucial context. The ethnic patterns we observed may therefore reflect inherent physiological differences, varied responses to PA, or the broader interplay of genetic, environmental, and socioeconomic factors known to shape pulmonary health disparities.

The beneficial effects of physical activity on lung function may extend beyond mechanical improvements. Evidence suggests that regular physical activity attenuates the decline in lung function in chronic conditions such as COPD, highlighting its potential to enhance respiratory muscle strength and reduce inflammation (48, 49). These findings align with our results, indicating that high-intensity activity may offer superior protective effects against respiratory decline, even in the adolescent population.

### 4.1 Strengths and limitations of the study

This study has several notable strengths. It utilizes data from the NHANES database, a large, nationally representative dataset encompassing diverse adolescent populations, which enhances the generalizability of the findings. The analysis focuses on both high- and moderate-intensity physical activities, providing detailed insights into their differential impacts on lung function. Furthermore, subgroup analysis highlights important demographic disparities, particularly racial differences, contributing to a deeper understanding of the factors influencing pulmonary health.

However, certain limitations should be noted. The cross-sectional design prevents the establishment of causal relationships, necessitating longitudinal studies for future exploration. The simplistic categorization of physical activity, based only on intensity and duration, overlooks variations in type and frequency, which could influence outcomes. Additionally, the study does not comprehensively address confounding factors such as genetic predispositions, environmental conditions, and socioeconomic status. Lastly, while the focus on adolescents provides valuable insights, the findings may not be generalizable to other age groups or populations.

## 5 Conclusion

This study highlights the significant role of physical activity, particularly high-intensity exercise, in improving lung function during adolescence. The findings underscore the importance of promoting physical activity as a key intervention for pulmonary health. Subgroup analysis further reveals racial disparities, emphasizing the need for targeted public health strategies. While limitations exist, such as the cross-sectional design and simplistic activity categorization, this study provides valuable insights into the relationship between physical activity and lung function. Future longitudinal research is needed to confirm causality and refine activity classifications, ensuring better lung health outcomes for adolescents.

## Data Availability

The original contributions presented in the study are included in the article/supplementary material, further inquiries can be directed to the corresponding authors.

## Footnotes

^1^ http://www.cdc.gov/nchs/nhanes.htm

^2^ https://www.statsape.com
